# KU124 (9,10,10-trioxo-N-(2-phenylphenyl)thioxanthene-3-carboxamide) as a novel inhibitor of TASK-1

**DOI:** 10.3389/fphar.2025.1577171

**Published:** 2025-06-25

**Authors:** Ana Dumani, Annette Jacob, Diego Chavez, Abena Amankwaa, Ramish Zahed, Martha Julemis, Hinaben Patel, Joana Lopez, Steven Almazan, Patrick Martins, Anthony Contreras, Sofiia Korotka, Gianna Kiszka, Alexander Aleynik, Justin Patino, David Graham, Megan Blaisdell, Youssef Elhowary, Shuayb Yousuf, Chelsea Pelley, Jenna Marciano, Jessica Best, Rhustie Valdizno, Nikki Mastrodomenico, Jonelle Brown, Sarah Schwartz, Irene Anin, Yara Farrag, Rinchu George, Gianna Medeiros, Sophia Lang, Marilyn Dennis, Oluwatoni Awoleye, Lamont Lee, Ericka Salgado, Diana Figueroa Chea, Thomas Walter Comollo

**Affiliations:** ^1^ Kean University, Union, NJ, United States; ^2^ Touro University, New York, NY, United States

**Keywords:** TWIK-related acid-sensitive potassium channel 1, virtual screening, inhibitor, KU124, molecular dynamics, thallium flux, ion channel, two-pore potassium channel

## Abstract

TASK-1 is a two-pore K^+^ leak channel. The name, TASK-1, stands for TWIK-related acid-sensitive potassium channel 1, and this channel is encoded by the *KCNK3* gene. TASK-1 channels are expressed in humans and modulate cell excitability in excitable cells such as neurons, cardiomyocytes, and vascular smooth muscle cells. TASK-1 inhibition is a mechanism of action for some respiratory stimulants, such as doxapram. TASK-1 channels have also been suggested to play a role in circumventing cell apoptosis in a population of non-small-cell lung cancer cells. We propose that the inner vestibule of the TASK-1 channel, a known binding site of known TASK-1 inhibitors, BAY10000493 and BAY2341237, can be exploited via virtual screening to find other novel TASK-1 inhibitors. Our results show that by targeting the inner vestibule site, we found an active TASK-1 inhibitor. We suspect that this region of interest can be further exploited to discover additional TASK-1 inhibitors. Our initial success lends validity to our virtual screening methodology and parameters. In this study, we identified a novel TASK-1 inhibitor, KU124, which we verified using an *in vitro* assay.

## Introduction

Two-pore potassium (K^+^) channels are K^+^-selective ion channels composed of potentially mixed dimers of α-subunits (Comollo et al., 2020). TASK-1 channels are membrane-spanning ion channels, providing a pathway for K^+^ to pass through the membrane. The TWIK-related acid-sensitive potassium channel 1 (TASK-1) is encoded by the *KCNK3* gene and is found in neurons, cardiomyocytes, and vascular smooth muscle cells (Comollo et al., 2020; Olschewski et al., 2017). TASK-1 channels are involved in the regulation of heart rate, pulmonary artery tone, sleep/wake cycles, and responses to volatile anesthetics. In the central nervous system, TASK-1 has its highest expression in the cerebellum (Fan et al., 2022). The cerebellum is known to control movement, but it also controls emotional and motivational states (Rudolph et al., 2023). The expression of TASK-1 outside the central nervous system, most pertinent to this work, is its expression in the lungs, pancreas, heart, and pulmonary arteries (Fan et al., 2022). At physiological resting potentials, TASK-1 channels are constitutively active at a low level in excitable cells, with their activity increasing when the cell depolarizes (Comollo et al., 2020; Olschewski et al., 2017).

TASK-1 inhibitors, such as doxapram, are already in use as ventilatory stimulants to stimulate the breathing center of the brain (Yost, 2006). By inhibiting TASK-1, the dependent neuron will become more excitable (Nicoll et al., 1990).

In atrial fibrillation, TASK-1, which is often upregulated, causes a shortening of the atrial action potential duration. Lengthening the cardiac action potential’s duration by inhibiting TASK-1 could provide a therapeutic effect on the patient’s atrial fibrillation. This effect on TASK-1 and atrial fibrillation is considered to be part of the therapeutic action of ranolazine (Ratte et al., 2019). The known TASK-1 inhibitor, doxapram, is also an effective antiarrhythmic in a porcine model of atrial fibrillation (Wiedmann et al., 2022). A293, an experimental TASK-1 inhibitor, is effective in rhythm control in a large animal model of atrial fibrillation (Flaherty et al., 2014).

TASK-1 channels have even been predicted to play a role in tumorigenesis and circumventing cell apoptosis in a population of non-small-cell lung cancer cells, and a reduction of TASK-1’s effect has been reported to reduce cancer cell proliferation (Leithner et al., 2016; Arévalo et al., 2022).

There are several potential clinical uses of TASK-1 inhibitors yet to be explored, as illustrated above.

Virtual screening is the process of performing a multitude of computational small-molecule docking simulations on a large catalog of drug-like compounds. It has been used to enrich pools of molecules for *in vitro* testing to identify potential drug candidates (Kaserer et al., 2016; Kimani et al., 2017; Manglik et al., 2016). By conducting virtual screenings consisting of millions of docking simulations, we can assess several drug-like small molecules—something that would be completely prohibitive with *in vitro* testing alone. This allows us to select the top-scoring compounds, or the “cream” of the screening, for further testing. These “hit” molecules are then subjected to *in vitro* evaluation. This approach saves valuable time and resources and increases the likelihood that the compounds we test will exhibit TASK-1-inhibiting activity. Our initially tested hit, KU124, represents a novel scaffold; the derivatives can be further screened for their efficacy in inhibiting TASK-1.

Crystal structures of TASK-1 released in 2019 show that the highly potent TASK channel inhibitors, BAY10000493 and BAY2341237, bind to an inner vestibule of the TASK-1 channel, where they become trapped by the channel’s lower X-gate upon the closing of the channel and stabilize the closed channel (Rodstrom et al., 2020). The high rate of attrition of molecules in the drug discovery process requires numerous starting lead molecules. By targeting the vestibule occupied by BAY10000493 in 6rv3.pdb and BAY2341237 in 6rv4.pdb, we have gained validity for our screening methodology for TASK-1 by discovering a novel and potent TASK-1 inhibitor, KU124.

## Materials and methods

### Virtual screening

Our current macromolecule model was created by adding the missing residues to chains C and D of 6rv2.pdb as they were detected in Academic Maestro 13. Glide was not used in this study because it is not included in the Academic Maestro 13 package available to us. We then filled in the missing residues’ side chains using Discovery Studio Visualizer by “mutating” each missing amino acid to the same type, thereby restoring its side chain. It is surmised that a similar model would have been obtained using chains A and B as both chains A and B and chains C and D comprise a complete TASK-1 pore-forming dimer.

**FIGURE 1:**
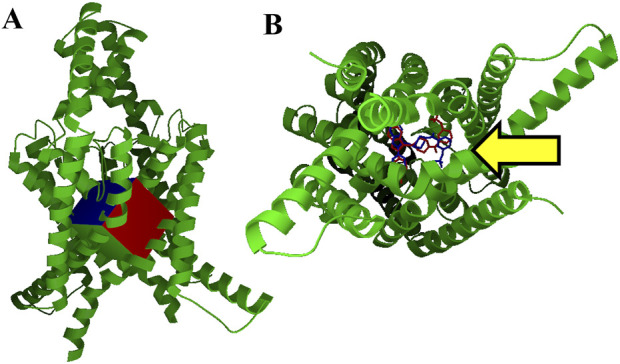
Virtual screening grid box and control docking **(A)**. Our TASK-1 macromolecule model (in green), used for virtual screening of ∼1 million ZINC database compounds, is pictured with the search area used for AutoDock Vina. This grid box encompasses the area of the vestibule occupied by the BAY series inhibitors found in 6vr3.pdb and 6vr4.pdb **(B)**. Intramembrane view of control docking (blue) and crystal structure conformation of BAY2341237 (red). BAY2341237 was docked into our apo TASK-1 macromolecule model using AutoDock Vina targeting the ROI in Figure 1A, which is pointed at by a yellow arrow (Vina score: −11.5 kcal/mol). The crystal structure positioning of BAY2341237 (magenta) was later juxtaposed into the figure to illustrate the similarities of the pose.

The macromolecule file was then prepared using AutoDock Tools by adding polar hydrogens, AD4 atom types, and Gasteiger charges. For our positive control, BAY2341237 was extracted from 6rv4.pdb and minimized it in Maestro 13. We then performed an *in silico* small molecule docking of BAY2341237 to the search grid box pictured in Figure 1 using AutoDock Vina 1.1. The search box used was tightly aligned with the BAY2341237 binding site to minimize false positives and ensure that only hit molecules fitting within the defined region of interest (ROI) were returned. For our current virtual screening, we utilized the same search grid box, targeting the ROI occupied by BAY2341237, and AutoDock Vina 1.1 (Trott et al., 2010). Our virtual screening was performed using a method that utilizes shell scripts to run AutoDock Vina in “for each” loops and uses other scripts to process files and sort hits. These shell scripts have been previously utilized to identify active biological modulators (Kimani et al., 2017). Examples of these scripts may be found available online at https://home.fatsilicodatapharm.com/home/virtual-screening-methodology.

Approximately one million compounds for this initial screening were selected from the ZINC12 (zinc12.docking.org) “clean drug-like” set. Candidates for *in vitro* testing were selected based on favorable Vina scores, appropriate conformations within the ROI, and compound availability.

#### Molecular dynamics

Molecular dynamics (MD) systems, including the KU124 ligand, were prepared in CHARMM-GUI (Jo et al., 2008; Wu et al., 2014; Brooks et al., 2009; Lee et al., 2016) and VMD 1.9.3 (Humphrey et al., 1996) using our docking models. The ligand, KU124, was prepared by importing the .pdb file into the CHARMM-GUI Ligand Modeler and using FF Gen and CSML Search (Jo et al., 2008; Kim et al., 2017). These systems have a CHARMM36 force field and were run in NPγT dynamics. The MD systems contained 0.15 M KCl and explicit H_2_O. Both systems were derived from an original system, and the model of KU124 was merged with the indicated model using VMD 1.9.3 (Humphrey et al., 1996). The two systems were energy-minimized, heated to 310 K, and then subjected to 1.5 µs of molecular dynamics simulation using NAMD 2.14 (Phillips et al., 2020) with the NPγT ensemble. Visualization was carried out in VMD (Humphrey et al., 1996). DeepView (Swiss PDB Viewer) (Guex and Peitsch, 1997) was used for the RMSD analysis. Workstation(s) equipped with Intel Xeon Gold series CPUs and NVIDIA RTX-series GPUs were used to run the CUDA-enabled version of NAMD 2.14.

#### Chemical synthesis and compound selection

Following virtual screening, three selected good-scoring candidates, including KU124, were purchased for experimental validation. These were purchased from Enamine (Ukraine), and their purity was confirmed by the manufacturer via mass spectrometry.

#### Cell culture

An inducible TASK-1-GFP-expressing Chinese hamster ovary (CHO) cell line (a generous gift from Dr. Douglas Bayliss at the University of Virginia) was used in this study. The base medium was prepared using Ham ’s-F12 with L-glutamine, supplemented with 10% FBS and 1% penicillin/streptomycin. Blasticidin and hygromycin B were added to apply selection pressure and create the selection growth medium for the TASK-1-GFP-inducible CHO system, at concentrations of 10 μg/mL and 200 μg/mL, respectively. The TASK-1-GFP CHO cells were maintained in the selection growth medium, while the base medium was used for cell splitting and preparation for the pre-thallium flux experiment.

#### 96-well plate preparation

The TASK-1-GFP CHO cells were seeded into a 96-well plate and either treated with the base medium containing tetracycline or left as untreated controls. In the tetracycline-treated wells, a final concentration of 125 μg/mL was used. Induction of TASK-1-GFP in the tetracycline-treated inducible TASK-1-GFP CHO cells was visually checked 24 h after splitting a confluent T25 flask (or an equivalent cell growth area) into a 96-well plate.

#### Thallium flux assay

After the cells were split and stimulated with tetracycline in the 96-well plate for 24 h, the plates containing the TASK-1-GFP CHO cells were used for thallium flux experiments. In some trials, entire plates were either treated with tetracycline or left untreated. In the majority of the trials, one half of the plate’s rows were treated with tetracycline, while the other half were left as untreated controls.

To begin the thallium flux assay, the media was aspirated, and 150 μL of PBS was added to each well in order to wash away the media and tetracycline. PBS was then removed, and 60 μL of thallium flex dye-loading solution was added to each well. Each 10 mL of dye-loading solution was prepared using 6 µL Brilliant Thallium indicator solution, 200 μL of Dysol, 1 mL of 10x assay buffer (from the ION Biosciences Kit), 200 μL of probenecid solution, and 8.6 mL of deionized water. This solution was prepared following the instructions provided with the Brilliant Thallium Flex Kit (ION Biosciences). The amount of dye used was slightly reduced from the prescribed amount to avoid saturation in the plate reader. The cells were then incubated at room temperature for 1–1.5 h. The wash solution was prepared by adding 1 mL of the ION Biosciences Kit’s 10x Assay Buffer and 9 mL of deionized (DI) water. The dye-loading solution was removed, and 60 μL of wash solution was added to each well. Then, 15 μL of the wash solution containing five times the desired final concentration of thallium was added to each appropriate well, resulting in a 1x concentration. This was then incubated for 20 min.

The thallium stimulus solution was prepared using 500 μL of 10x chloride-free stimulus buffer, 500 μL of 10x high-potassium stimulus buffer, 500 μL of a 50 µM thallium sulfate solution, and 8.5 mL of DI water, following the Brilliant Thallium Flex Kit instructions for a final volume of 10 mL. A baseline reading of fluorescence was carried out first using a Tecan plate reader at an excitation wavelength of ∼490 nm and an emission wavelength of ∼520 nm. An amount of 60 μL of solution 4 was quickly co-pipetted using a 12-channel multichannel pipettor onto each row of wells, ensuring the same time-point across each row. This was then quickly read in the plate reader; the process of pipetting to read took approximately 30 s. The kinetic reading of fluorescence was recorded at 30 s intervals for an additional 720 s for a total read time of 750 s.

A final concentration of 10 µM doxapram, a known TASK-1 inhibitor, was used as a comparative control. All conditions being tested were run in at least four sets of triplicate samples across four separate rows on each plate that were analyzed and then averaged to achieve one trial (n = 1) (Figure 5).

### Thallium flux data analysis

Microsoft Excel software was used to analyze data from thallium flux assays from the spectrophotometric reader TECAN 590. Data were calculated from the expressed average between the triplicates from each component. Each reading for each well was divided by the initial baseline reading to yield a fold change. The average of the drug triplicate fluorescence was subtracted from the average of the two vehicle controls to yield a drug-sensitive fold change. Each half dish (four rows) or full dish (eight rows) of tested tetracycline-induced wells was averaged as one trial (n = 1). The KU124 dose-response curve was generated in Origin 2021b by fitting the data to a Hill equation.

## Results

### Virtual screening yields hit molecules

Our TASK-1 macromolecule model was created as described from chains C and D of Protein Data Bank entry 6rv2.pdb. For all dockings and virtual screenings, we used the search grid box pictured in Figure 1A using AutoDock Vina. Figure 1B shows our control BAY2341237 docking in blue, which is juxtaposed against the crystal structure conformation in red. Virtual screening was performed using shell scripts to run AutoDock Vina in “for each” loops, along with other scripts for data management.

**FIGURE 2:**
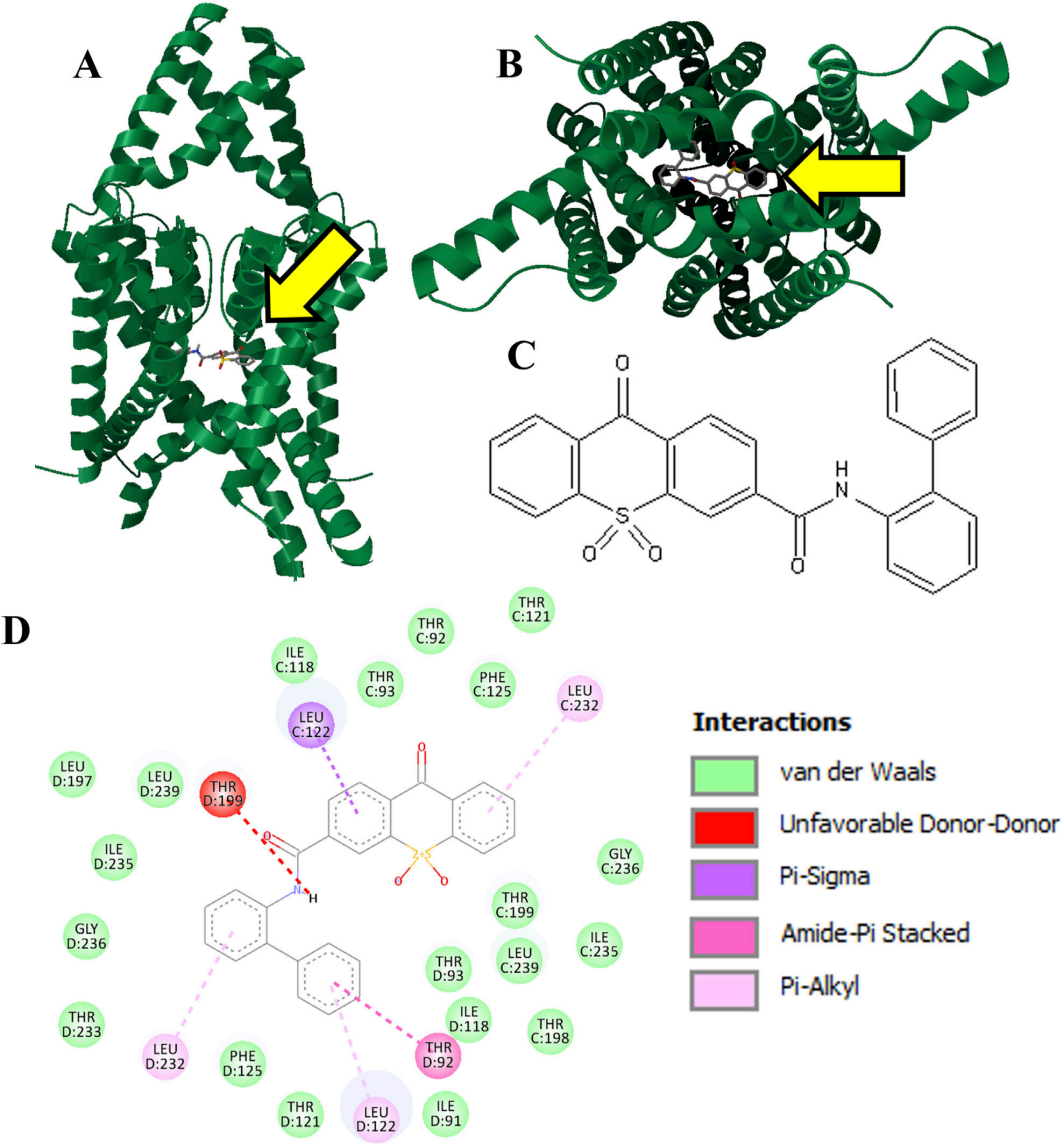
Hit molecule KU124 (Vina score: −13.1 kcal/mol). KU124 was docked into our apo TASK-1 macromolecule model using AutoDock Vina, targeting the ROI in Figure 1A, which is also pointed at by a yellow arrow **(A).** Transmembrane view **(B).** Intramembrane view **(C)**. 2D chemical structure of KU124, which is ZINC entry ZINC15643722, with the chemical name (9,10,10-trioxo-N-(2-phenylphenyl)thioxanthene-3-carboxamide) **(D).** Residue interactions as calculated by Discovery Studio Viewer.

We were able to obtain selected members of the top Vina scoring from the ∼1 million molecules screened for testing. Three hit molecules were selected for purchase and *in vitro* evaluation. The most successful molecule was KU124, the docking of which is shown in Figures 2A, B. The structure for KU124, a novel compound for TASK-1 inhibition, is depicted in Figure 2C. KU124 corresponds to the ZINC database entry ZINC15643722, with the chemical name (9,10,10-trioxo-N-(2-phenylphenyl)thioxanthene-3-carboxamide). KU124-predicted interacting residues are shown in Figure 2D.

### The MD models adjusts to different conformations

**FIGURE 3:**
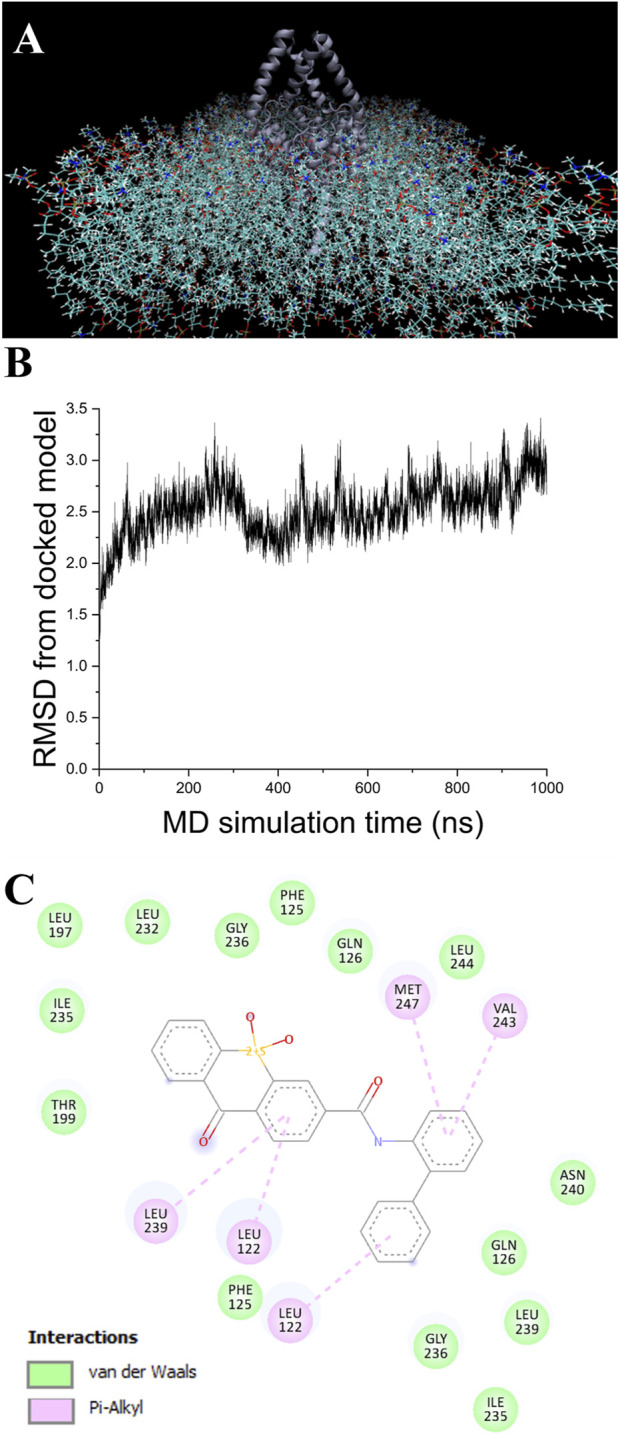
MD simulation. **(A)** Model of TASK-1 (center dark blue), with KU124 docked and observed in the POPC membrane. Explicit H_2_O and KCl were also used (not shown) **(B).** RMSD from the original docked model, as calculated over 1 µs of NAMD NPγT molecular dynamics simulation using a CHARRM36 force field **(C).** KU124 interacting with TASK-1 residues after 1.5 µs of NAMD NPγT molecular dynamics simulation using a CHARMM36 force field.

A single 1.5 µs MD simulation using NAMD 2.4 MD was performed for each of the apo and the KU124-bound TASK-1 models. The KU124-bound system is shown in Figure 3A.

In the first 100 ns, the KU124-bound model underwent a rapid adjustment, which then stabilized (Figure 3B) to a conformation that averaged 2.56 Å RMSD from the starting conformation over the next 900 ns. After 1.5 µs, a comparison of the pre-dynamics model’s backbone vs. that of a 1.5 µs of NPγT NAMD molecular dynamics with the KU124 docked model was 3.10 Å.

**FIGURE 4:**
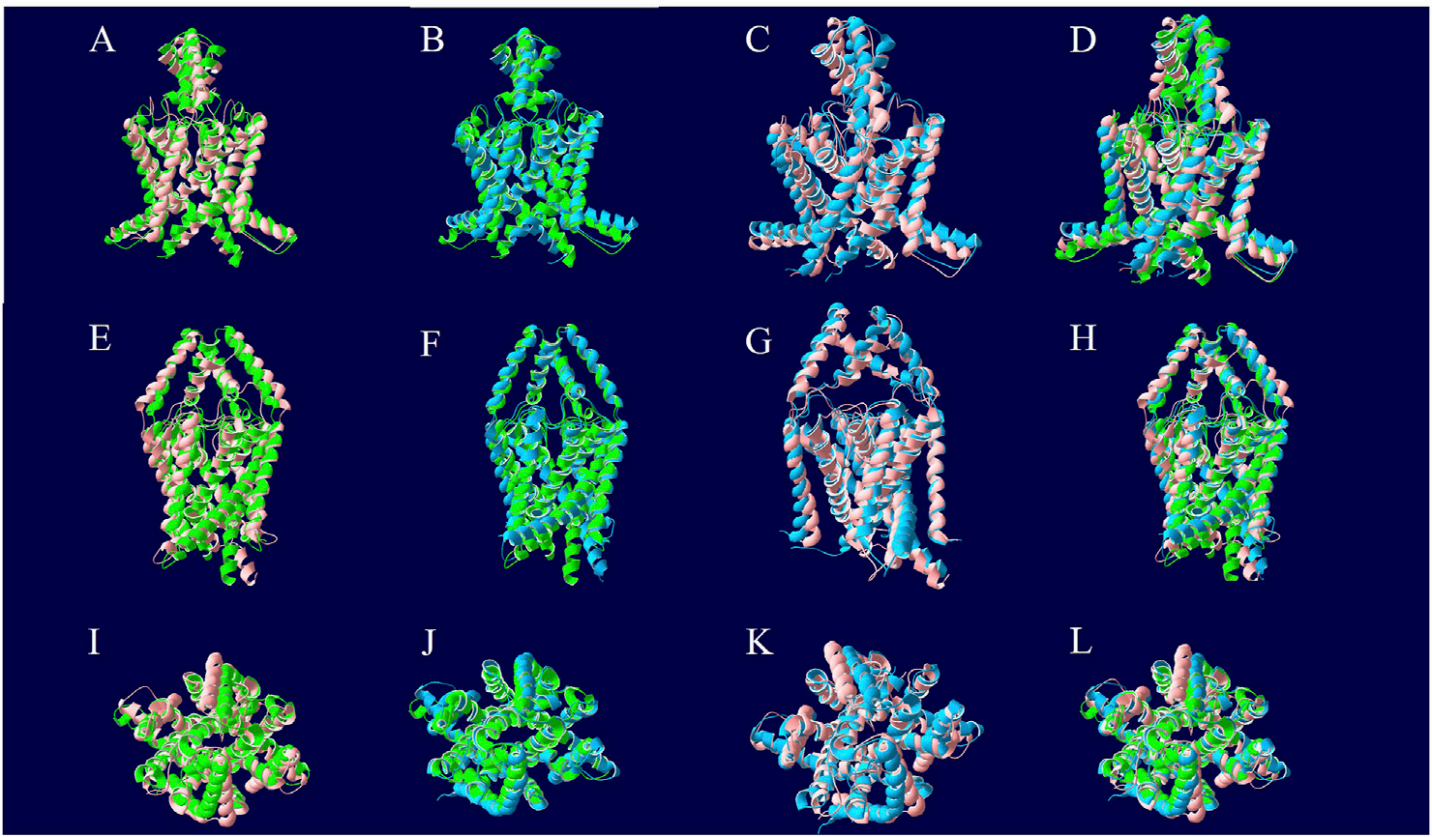
Structural alignment of TASK-1 MD models. The 0 ns model is depicted in green and was the beginning point for both molecular dynamics runs. The protein of the KU124-bound model after 1.5 µs of MD simulation is in blue. The 1.5 µs apo model is red. The models were aligned along their backbone in DeepView using “Magic Fit.” The top row is the first intramembrane angle shown for each structural alignment. In the middle row, we see intramembrane rotated approximately 90° around the Y-axis. The bottom row is the extracellular pore view. **(A, E, and I)** The 0 ns TASK-1 model aligned along backbone with the 1.5 µs apo model. **(B, F, and J)** The 0 ns TASK-1 model aligned along backbone with the 1.5 µs KU124-bound model. **(C, G, and K)** The 1.5 µs apo and KU124-bound MD models aligned along their backbones. **(D, H, and L**) All three models; 0 ns, 1.5 µs apo, and 1.5 µs KU124-bound model, aligned on their backbones.

A comparison of the apo pre-MD model of TASK-1 (0 ns) to the model after 1.5 µs of NPγT NAMD 2.4 molecular dynamics with explicit solvation showed a backbone RMSD of 3.07 Å. Comparing that same pre-dynamics model’s backbone to that of the model after 1.5 µs of NPγT NAMD 2.4 molecular dynamics with KU124 docked yielded an RMSD of 3.10 Å. While at first glance this may suggest that the two 1.5 µs conformations are very similar, the RMSD between the bound and unbound 1.5 µs conformations is 3.48 Å, indicating notable differences. Taken together, these results suggest that the crystal structure lies in an intermediate state between those induced by the dynamics in the apo form and the KU124-bound form. Thus, the conformations of the apo- and KU124-bound dynamics models are different. A pictorial depiction of the structural alignment of these three models is shown in Figure 4.

KU124 remains in TASK-1 throughout the bound MD simulation. At the end of the 1.5 µs-bound MD simulations, the KU124 small molecules adopt a conformation lacking the clashing interaction with THR199 (Figures 2D, 3C). LEU122 appears to be the most consistent contributor to KU124 binding, with predicted Pi–alkyl bonds to aromatic rings at both time-points, as predicted by Discovery Studio Viewer (Dassault Systèmes) (Figures 2D, 3C).

#### KU124 shows dose-dependent TASK-1 inhibition *in vitro*

**FIGURE 5:**
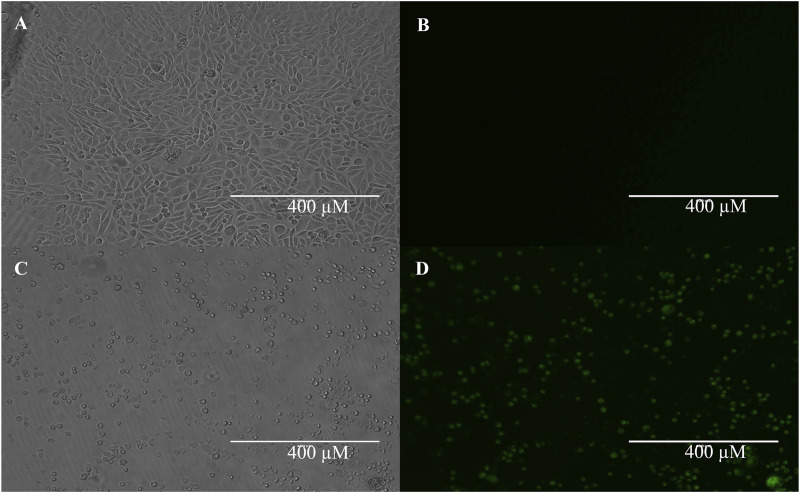
TASK-1-GFP expression in tetracycline inducible cells. TASK-1-GFP CHO cells with no tetracycline induction show little-to-no expression of TASK-1 GFP **(A)** brightfield; **(B)** fluorescent). TASK-1-GFP CHO cells with 24 h 0.125 mg/mL tetracycline induction show robust TASK-1-GFP expression **(C)** brightfield; **(D)** fluorescent).

**FIGURE 6:**
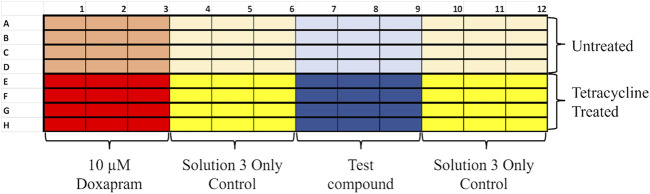
Example layout of a thallium flux 96-well plate. Plates were divided into rows to be co-pipetted; each row had its own controls to compare to. Half of the plate would be the untreated control, and the other half was treated with tetracycline to induce TASK-1 expression. Each row had a positive for TASK-1 inhibition control (10 µM doxapram) set of triplicate samples. Doxapram is a known inhibitor of TASK-1 [12]. Each row also had two vehicle-only triplicates to ensure a good baseline to subtract doxapram sensitivity and KU124 triplicates at the concentration being tested.

Figure 5 shows our TASK-1-GFP CHO cells growing in culture in parts A (brightfield) and B (fluorescence). In parts C (brightfield) and D (fluorescence), we demonstrate our ability to induce TASK-1-GFP in the TASK-1-GFP CHO cells. Tetracycline was used to induce the TASK-1 channel protein expression. Tetracycline-induced cells showed a visible increase in these cells’ fluorescence in a clear 96-well plate. These cells were also used for a previously published study seeking TASK-1 inhibitors and are shown in Figure 6 (Widemann et al., 2022). Our thallium flex protocol results in significantly greater fluorescence compared to the baseline in the tetracycline-induced cells (data not shown).

**FIGURE 7:**
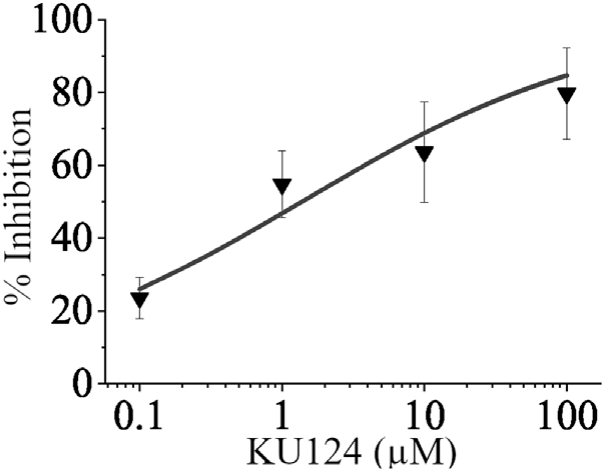
KU124 inhibits TASK-1 in a dose-dependent manner. Dose-response curve showing TASK-1 inhibition by KU124 in a TASK-1 expressing cell line. A concentration of 10 µM doxapram, a known TASK-1 inhibitor, was used as a positive control. Each concentration point represents n≥4.

Figure 7 shows the dose-dependent inhibition of fluorescence in the form of a dose-response curve in which the TASK-1-expressing cell line’s thallium conductance was inhibited by KU124. An amount of 10 μM of doxapram, a known TASK-1 inhibitor, was used as a positive control. Our predominant 96-well plate setup is shown in Figure 5. Each point on our dose-response curve consists of the average of four or more independent 96-well plate trials. KU124’s extrapolated IC_50_ value is 1.38 ± 0.652 µM.

## Discussion

Leveraging computational tools, such as AutoDock Vina and utilizing structural data from Protein Data Bank (PDB) entries 6RV2, 6RV3, and 6RV4, we demonstrated the ability to efficiently target the inner vestibule of TASK-1 channels to identify novel inhibitory compounds. This approach allowed us to screen approximately one million commercially available drug-like molecules from the publicly accessible ZINC12 database (zinc12.docking.org). This is something that, for us, would have been prohibitively time-consuming and resource-intensive using traditional *in vitro* methods alone. This approach saves valuable time and resources and ensures that the majority of the hits that we test *in vitro* are more prone to have some TASK-1-inhibiting activity. Additionally, our initially tested hit, KU124, represents a novel scaffold, the derivatives of which can be further screened *in silico* and *in vitro*.

In the original Rodstrom paper corresponding to the TASK-1 crystal structures used, the channel structure is presented as being in a closed, non-conducting state. In molecular dynamics simulations, our modeling appears to induce a distinct KU124-bound TASK-1 channel conformation that differs from the crystal structure and is, presumably, non-conducting. Meanwhile, the apo model adopts a different conformation, possibly representing a transition to yet another state. The RMSD comparisons across conditions, including the 3.48 Å backbone deviation between the final states of the apo- and KU124-bound models, further suggest that TASK-1 undergoes a ligand-induced shift rather than merely fluctuating around a single equilibrium state. Although these findings must be acknowledged with caution due to the n = 1 nature of the MD section of our study, taken together, these findings provide structural evidence that TASK-1 exhibits a dynamic response to ligand binding, with implications for its gating and functional regulation.

TASK-1 is implicated in various physiological and pathological conditions due to its role in maintaining K^+^ ion homeostasis. The ability of KU124 to inhibit TASK-1 activity, as demonstrated in this study, suggests its potential therapeutic application across a range of diseases. KU124 may possess therapeutic value in atrial fibrillation, cardiac dysfunction, depression, and other conditions, which is supported by data from this study and the relevant literature.

Figure 6 of this manuscript illustrates the dose–dependent TASK-1 inhibition achieved by KU124.

TASK-1 channels are critical regulators of atrial repolarization. Overexpression or dysregulation of TASK-1 has been associated with atrial fibrillation, a prevalent cardiac arrhythmia (Schmidt et al., 2015). By modulating atrial action potentials, TASK-1 inhibitors such as KU124 could possibly stabilize electrical activity and reduce AF episodes.

TASK-1 is also expressed in the central nervous system, where it plays a role in regulating neuronal excitability and neurotransmitter release. The selective inhibition of TASK-1 has been predicted as a potential strategy for the treatment of depression (Fan et al., 2022; Bayliss et al., 2008). KU124 may be used to restore less pathological neuronal activity, offering a novel approach to depression management. TASK-1 expression in the cerebellum leads to a hypothesis that a TASK-1 inhibitor could be used as a motivational, alertness, and awakeness aid. This may be useful to military personnel and first responders in times of crisis. Additionally, TASK-1 inhibition has shown potential in neuroprotection by reducing ischemia-induced neuronal injury (Meuth et al., 2009).

From an oncological perspective, TASK-1 channels have been suggested to play a role in the evasion of apoptosis in non-small-cell lung cancer cells (Leithner et al., 2016; Arévalo et al., 2022). TASK-1 knockdown has been associated with increased apoptosis rates, suggesting that pharmacological inhibition could sensitize malignant cells to therapies such as CAR-T and CAR-NK therapies. In this context, if this is true, then TASK-1 inhibitors such as KU124 could potentially serve as adjuvant therapies, enhancing the cytotoxic effects of immune therapies and improving treatment outcomes in resistant cancer phenotypes. If inhibiting TASK-1 leads to disrupting a TASK-1-mediated apoptotic resistance, this would add another layer of therapeutic versatility to these inhibitors.

KU124 will have to be further tested to better characterize its effect on TASK-1, such as via patch clamp tests, and observe its effects on other ion channels. KU124 may have an effect on TASK-3 and should also be verified for its effect on hERG, for example. These would be excellent future steps to be carried out by our group or another group.

IPSC-derived cardiomyocytes (Comollo et al., 2022) and neurons (Farkhondeh et al., 2019) have successfully been used to test pharmacological agents’ effects and can be used to further characterize KU124 using patch clamp techniques. Future studies should also explore the combinatory effects of TASK-1 inhibitors with existing drugs for synergistic benefits.

Although the current study establishes KU124 as a potent TASK-1 inhibitor, further investigation is required to assess its safety and efficacy *in vivo*. Structural optimization and pharmacokinetic studies will be essential for translating these findings into clinical therapies.

Our virtual screening parameters defined a very tight search area around the binding sites of BAY10000493 and BAY2341237. This approach was carried out to minimize false positives that can arise from using an overly broad search area, which might yield high scores for binding to a site other than the functional site. The validation of our virtual screening parameters through the identification of KU124 shows that we may be able to find additional, perhaps more useful, TASK-1 inhibitors using these methods.

## Data Availability

The original contributions presented in the study are included in the article/supplementary material; further inquiries can be directed to the corresponding author.
